# Metformin exerts synergistic anti-proliferative effects with liraglutide in human umbilical vein endothelial cells (HUVECs)

**DOI:** 10.22038/IJBMS.2022.64117.14117

**Published:** 2022-04

**Authors:** Amir Shadboorestan, Samane Eftekhari, Negar Mottaghi- Dastjerdi, Rezvan Shahparvari, Parastoo Tarighi, Hoda Jahandar, Homa Faghihi, Hamed Montazeri

**Affiliations:** 1 Department of Toxicology, Faculty of Medical Sciences, Tarbiat Modares University, Tehran, Iran; 2 Department of Medical Biotechnology, Faculty of Allied Medicine, Iran University of Medical Sciences, Tehran, Iran; 3 Department of Pharmacognosy and Pharmaceutical Biotechnology, School of Pharmacy, Iran University of Medical Sciences, Tehran, Iran; 4 Pharmaceutical Sciences Research Center, Tehran Medical Sciences, Islamic Azad University, Tehran Iran; 5 Department of Pharmaceutics, School of Pharmacy, Iran University of Medical Sciences, Tehran, Iran; # These authors contributed equally to this work

**Keywords:** Angiogenesis, Cancer, HUVECs, Liraglutide, Metformin

## Abstract

**Objective(s):**

Metformin (Met) and liraglutide (Lira) have been approved to treat type 2 diabetes mellitus and have cardioprotective effects.

**Materials and Methods:**

Human umbilical vein endothelial cells (HUVECs) were incubated with Met, Lira, or their combination in this study.

**Results:**

Results showed that the synergistic inhibitory effect of the two drugs on HUVECs proliferation was significant (75%) after 48 hr drug exposure. In addition, either Lira or Met alone had a marked tendency to inhibit the migration of HUVECs (42% and 39%). Almost a complete inhibition (97%) was demonstrated in combinational use after 48 hr treatment. After combining these two drugs, the apoptosis rate raised to 68%, which was a significant approval of synergistic apoptosis induction of Met and Lira. The combinational group indicated a substantial increase in VEGF, PDGF, and MMP-9 at 24 hr compared with the control.

**Conclusion:**

This study showed that combination therapy with Lira and Met could effectively reduce cell proliferation, induce apoptosis, and inhibit cell migration in the HUVECs. This study provides evidence to support using Met in combination with Lira as a treatment option for patients with type-2 diabetes and cancer.

## Introduction

Cancer, as a class of diseases characterized by uncontrolled and abnormal cell growth, demands a rapidly developed new vascular network, termed angiogenesis. Angiogenesis aids in supplying nutrients and oxygen for high proliferative cancer cells and clears their metabolic wastes, resulting in necrosis and apoptosis prevention (1, 2). Therefore, regulating vascular formation can be a target for cancer therapy. 

Pre-existing underlying diseases can affect the risk of onset and mortality of cancers. The International Agency for Research on Cancer (IARC) has reported that the incidence and progression of many cancers are strongly associated with diabetes. They have approved the association between insulin and cancer because hyperinsulinemia induces proliferative tissue abnormalities through DNA synthesis stimulation (3, 4). Furthermore, studies have introduced type 2 diabetes mellitus (T2DM) as an important risk factor for heart diseases, increasing the risk of cardiovascular death up to six folds (5, 6). Prescription of Met has been the most common treatment strategy for diabetes. The FDA has recently approved Lira, a GLP-1R analog, to treat type 2 diabetes, called “Incretin-based therapy” (7, 8). 

Met as the first-line treatment modality, suppresses hepatocyte gluconeogenesis and inhibits mitochondrial enzymes. The evidence indicates that patients with type 2 diabetes who take Met have a reduced risk of cancer compared with patients not taking it (9). In addition, more recent studies have reported the strong potential of Metformin in treating vascular dysfunction beyond diabetes, for its anti-inflammatory and anti-angiogenesis effects. Which accordingly, is suggested as the proposed action of Metformin in tumor suppression (10-12).

On the other hand, Lira applies its biological roles by binding to the GLP-1 receptor, widely expressed on pancreatic and extrapancreatic cells. It targets insulin secretion, glucagon release, and gastric emptying, leading to weight loss effectiveness (13). Several studies indicate that GLP-1 receptor agonists such as exendin-4 and liraglutide have anti-inflammatory effects and decrease cardiac and vascular inflammation (14, 15). Also, other protective effects in human vascular endothelial cells contributed to attenuating impacts of GLP-1 receptor agonists in reducing plasminogen activator inhibitor type-1 (PAI-1) and vascular adhesion molecule (VAM) expression (16).

Anti-cancer properties through ERK-MAPK inhibition and cardiovascular protective effects via JAK and STAT phosphorylation are also important functions of Lira and diabetes control (17, 18). Although there is controversial evidence suggesting that Met applies its cardio-protective and tumor suppression effects through inhibiting neovascularization, at the same time, Lira acts as a kind of pro-angiogenic factor. Nevertheless, they both are known as anti-tumors (10, 14). Therefore, concerning the mentioned critical role of angiogenesis in the progression of tumors, and the circular association between diabetes, vascular diseases, and cancer, we aimed to examine the synergistic effects of Met and Lira in concomitant use. For this purpose, we designed our study of their effects on HUVECs, acting as a model of vascular endothelium, which reportedly plays an irreplaceable role in driving angiogenesis (5).

## Materials and Methods

***Cell culture***

HUVE cells from Pasteur Institute Cell Culture Collection (Tehran, Iran) were cultured in HUVECs specified medium. The cell culture condition was optimized at 37 °C in a 5% CO_2 _humidified incubator. The medium was supplemented with 10% Fetal bovine serum (FBS) and 100 IU/ml penicillin, 100 μg/ml streptomycin, and changed every two days. 80% confluent when the cells reached %80 confluencies, they were passaged using 0.25% trypsin/EDTA. 

***Cell viability determination***

Cell proliferation and viability were evaluated by (4,5‐dimethylthiazol‐2‐yl)‐2,5 diphenyl tetrazolium bromide (MTT) (Sigma, Poole, Dorset, UK) assay as was described before (19). Briefly, HUVECs cells were seeded at the density of 10 × 10^3^ cells/well per 100 µl medium in 96 well cell culture plates at 37 °C. After 48 hr, the cells were treated with 0, 1, 3, 6, 10, and 20 mM of Met or 0, 1, 10, 20, and 40 µM of Lira alone or the combination of drugs for co-treatment. At 24 and 48 hr time intervals, the medium was removed and renewed with 100 µl fresh medium containing 20 µl MTT solution (5 mg/ml in PBS) and then incubated for an additional four hours. Subsequently, 100 µl Dimethyl sulfoxide (DMSO) (Merck) was added to each well to resolve the formazan crystals. The optical density was measured using a plate reader (Bio-Rad, Model 680) at 570 nm.

***Median effect analysis***

To determine the interactions of Lira and Met based on the synergistic/antagonistic activities, as was described before, the data from the cell viability assay was assessed through Compusyn software which is developed according to the Chou and Talalay model. The software specifies a combination index (CI) for each drug interaction and divides them into 3 groups; CI<1, CI=1, and CI>1, representing synergy, additive, and antagonism effects. The combination results were compared with the cytotoxicity of each drug alone.

***Wound healing assay***

HUVECs were seeded in a six-well plate at a density of 5×10^5 ^cells/well. After 36 hr, the medium was removed, and cells were starved with a fresh medium containing 1% FBS and incubated for 12 hr. When the cells reached 80-90% confluency, the monolayer was scratched with a sterile yellow (100 µl) pipette tip, as uniformly and straight as possible (20). Cells were washed twice with PBS to remove debris and exposed to 1 µM Lira, 13 mM Met, or a combination of 1 µM Lir + 13 mM Met. The plates were photographed at indicated time points (0, 24, and 48 hrs.), and the closure of the scratch was assessed using ImageJ. Each value is derived from three randomly selected fields.

***Apoptosis assay***

We used the Annexin V-FITC Apoptosis Detection Kit (640945, Bio legend) to evaluate apoptosis. According to the manufacturer’s instructions. Briefly, a density of 5×10^5 ^HUVECs was seeded and cultured in a six-well plate for 48h at 37 °C. Afterward, cells were treated in 3 groups; 1 µM Lira, 13 mM Met, and their combination as 1 µM Lira+13 mM Met. There was also an untreated group applied as a control. After 48 hr of drugs exposure, the cells were harvested by trypsin and centrifuged, and then the pellet was washed in cold PBS twice. Subsequently, the obtained cell pellet was re-suspended in 500 µl 1X Binding buffer. Later, the same amount of Annexin V-FITC and Propidium Iodide (5 µl) was added, mixed gently, and incubated at 4 °C in the dark for approximately 15 min. Next, the cells were pelleted by adding 1 ml of 1X binding buffer and centrifugation at 1200 rpm for 5 min. Eventually, the pellet was re-suspended in 500 µl of binding buffer and was read by BD FACS Calibur flow cytometer (BD bioscience, San Jose, CA, USA) and processed using FlowJo-v 7.6.1 software. 

***Western blotting***

The protein levels of VEGF, angiogenin, PDGF, MMP-9, and β-actin were determined through western blotting based on the protocol described in our previous study. Briefly, at 48 hr of drug exposure, cells were lysed by RIPA lysis buffer containing protease inhibitors, and protein concentrations were determined using a Bicinchoninic acid kit. After SDS-PAGE as a protein separation step, they were transferred into a PVDF membrane followed by incubation with 5% non‐fat dry milk buffer for 2 hr. After that, membranes were probed with specific primary antibodies (Cell signaling, MA, USA) at 4 °C overnight, rinsed with TBS+0.1% Tween 20, and subsequently incubated with conjugated goat anti-mouse/rabbit horseradish peroxidase (Bio-Rad, USA) for 1 hr at room temperature. The blots were developed using the BM chemiluminescence western blotting kit (Roche, Germany), and densitometry quantification was measured using ImageJ software (NIH, USA).

## Results

***Inhibitory effect of metformin and liraglutide on HUVECs proliferation, alone and in combination***

**Figure 1:**
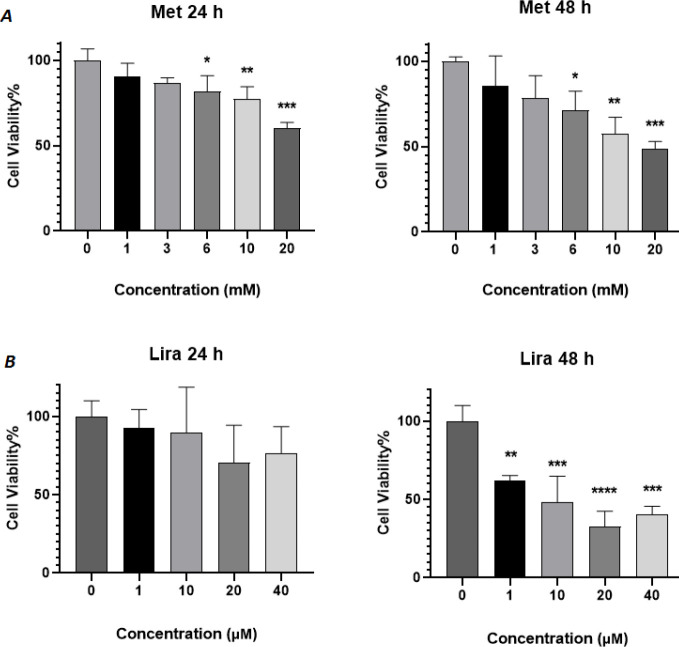
Viability of human umbilical vein endothelial cells (HUVECs) treated with A) Metformin and B) Liraglutide for 24 and 48 hr. Absorbance values were normalized to the control group (Mean ± SEM, n≥3). *P<0.05, **P<0.01, ***P<0.001, and ****P<0.0001

**Figure 2:**
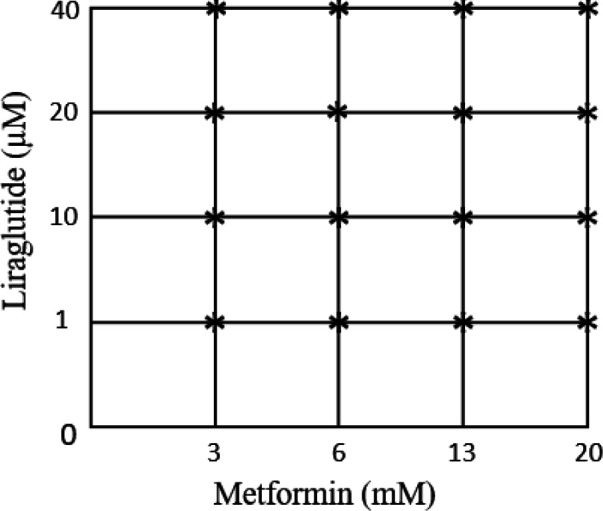
Matrix pattern for combining Metformin and Liraglutide. The marked plots are combinational doses

**Figure 3:**
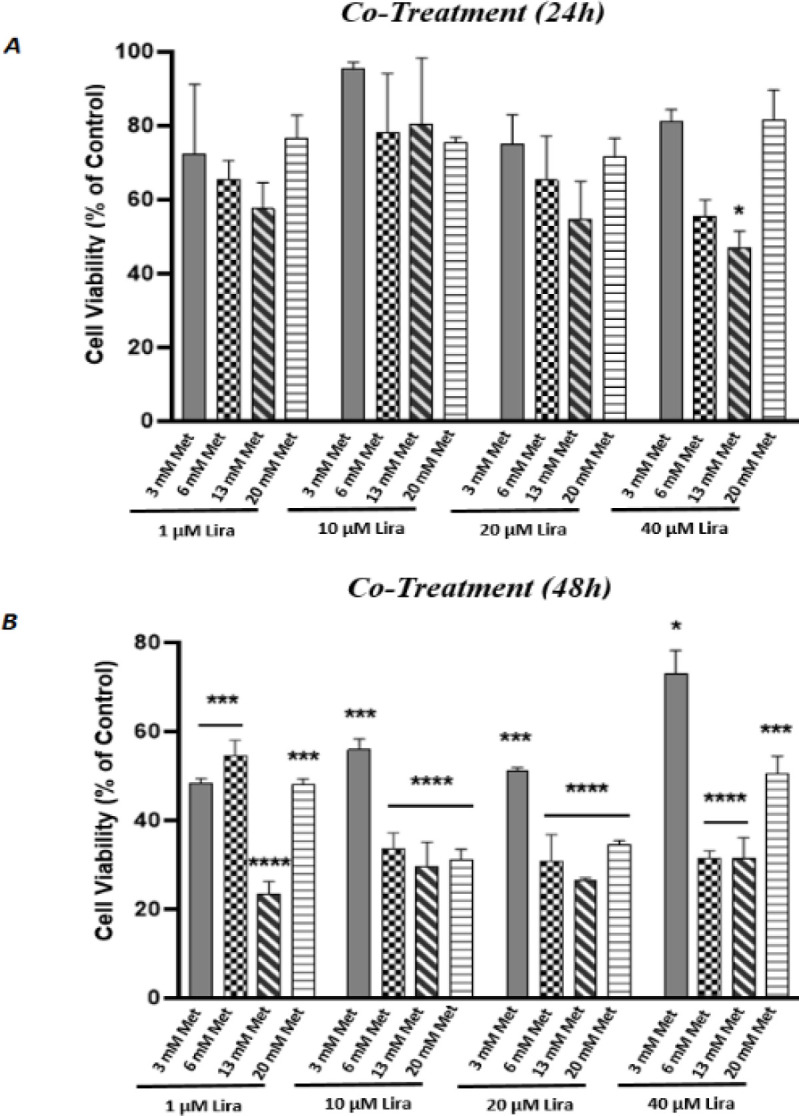
human umbilical vein endothelial cells (HUVECs) viability after being exposed to the combination of Metformin and Liraglutide after A) 24 hr and B) 48 hr, quantified by (4,5‐dimethylthiazol‐2‐yl)‐2,5 diphenyl tetrazolium bromide (MTT) assay. *P<0.05, ***P<0.001, ****P<0.0001 compared with control group

To assess the anti-proliferative effects of Lira and Met on HUVECS cells, we performed the MTT assay at 24 and 48 hr time intervals. Each drug independently showed significant dose-dependent growth inhibition. As shown in Figure 1 both Lira and Met indicated cytotoxicity with increasing dose and time. According to the outcomes, half IC_50_ was defined for each drug as 1 µM and 13 mM for Lira and Met, respectively. Then a range of low and high concentrations of each drug was selected and designed for co-treatment based on a matrix pattern (Figure 2). While the combination of Lira and Met at both time intervals, 24 and 48 hr, was cytotoxic, the synergistic inhibitory effect was significant after 48 hr drug exposure (Figure 3). Notably, 13 mM of Met concentration indicated the most growth inhibition in all combinational groups, showing up to 75% inhibition when combined with 1 µM Lira. 

***Synergistic interaction of Lira and Met***

**Figure 4:**
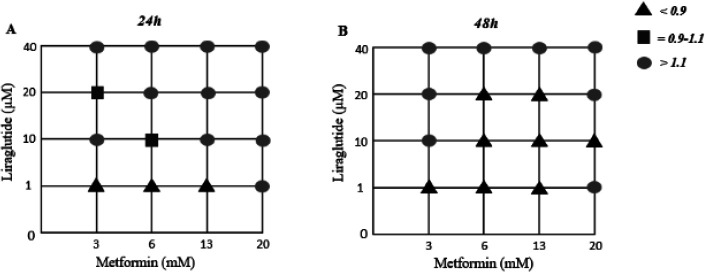
Synergy map compiling combination index (CI) values. The dots indicate synergism (CI<1), additivity (CI=1), and antagonism (CI>1) between Metformin and Liraglutide in human umbilical vein endothelial cells (HUVECs)

**Figure 5:**
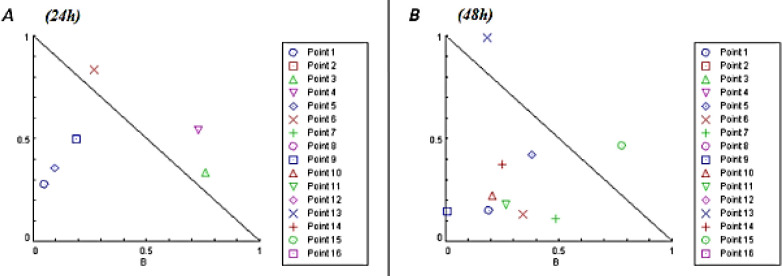
Isobologram diagram representing synergistic effects of Metformin and Liraglutide in A) 24 hr and B) 48 hr time intervals. The points under the diagonal line suggest the groups have synergistic activity

The CI values were assessed to determine the two drugs’ interaction in terms of synergism, antagonism, and additive effect. Cells were exposed to both drugs simultaneously for 24 hr and 48 hr. Figures 4 and 5 indicate the acquired synergy maps. At 24 hr analysis, 3 status of combinations revealed synergistic interactions (CI<1) while there was more synergism activity after 48 hr drug exposure, up to 8 groups. According to the results, we chose the combinational group of 13mM Met + 1 µM Lira for further experiments, in which both time intervals showed an acceptable synergistic index. The CI values are also listed in Tables 1 and 2; the synergistic effect groups are marked with *. 

***HUVECs migration inhibition through combined treatment with Lira and Met***

**Figure 6:**
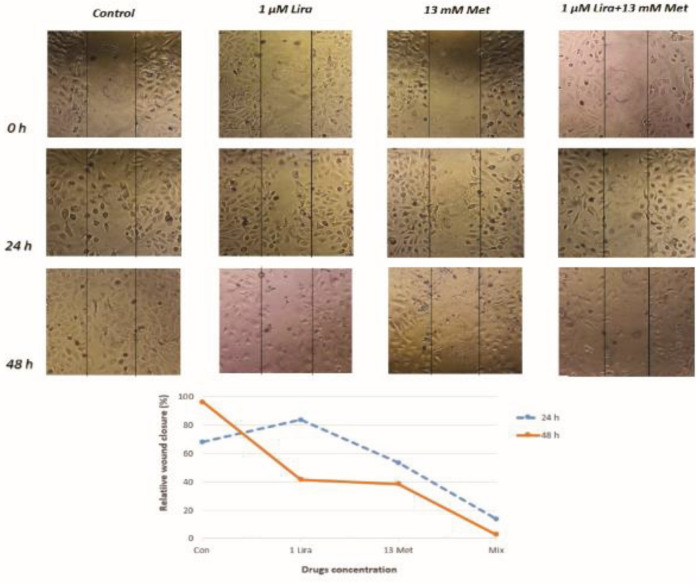
Migration of HUVECs treated with Metformin and Liraglutide alone and in combination. A wound-healing assay was performed to analyze cell migration, shown by either photograph (up) or histograms (down)

Since the migration of endothelial cells is known as a hallmark of angiogenesis, we assessed the HUVECS cell motility through wound healing assay at 24 and 48 hr intervals. Cells were treated with the IC_50_ of Lira and Met alone and in combination (1 µM Lira + 13 mM Met). After 24 hr, as shown in Figure 6, 1 µM Lira increased cell motility (83%) compared with control (68%), insignificantly. However, 13 mM Met decreased cell migration to 53.3% and 13% alone and combined with 1 µM Lira. When cells were exposed to drugs for 48 hr, either Lira or Met alone had a marked tendency to inhibit cell motility (41.6% and 38.5%). Compared with the control group in which the wound was totally filled by cells, almost a complete inhibition was demonstrated in the combinational group, indicating the inhibition of wound repair up to 97.2 %. 

***Apoptosis induction in HUVECs by Lira and Met synergistically***

**Figure 7:**
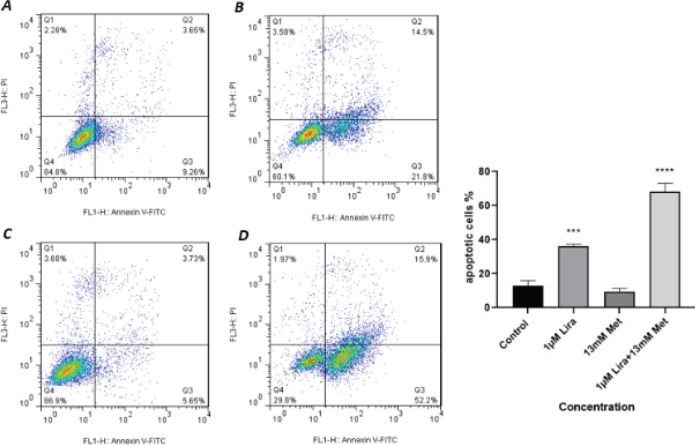
Apoptosis stimulation with Metformin and Liraglutide was shown by the percentage of apoptotic cells, analyzed by flow cytometry assay. Cells were divided into 4 groups, A) control, B) 1 µM Liraglutide, C) 13 mM Metformin, and D) combination of 1 µM Liraglutide +13 mM Metformin, incubated for 48 hr and stained with Annexin V-FITC and propidium iodide. Data are shown as mean ± SEM, n=3. (***P<0.001 and ****P<0.0001)

Apoptosis analysis was performed after 48 hr by Annexin V-FITC and PI staining with flow cytometry technique to investigate whether these drugs induce apoptosis in HUVECS cells. The test was conducted in 4 groups; control, 1 µM Lira, 13 mM Met and the combination of 1 µM Lira + 13 mM Met. As shown in Figure 7, a single treatment with 13 mM Met did not affect apoptosis contrary to the control group, neither in early apoptosis (Annexin V+/PI-; 5.56%) nor late apoptosis/necrosis (Annexin V+/PI+; 7/41%). However, 1 µM Lira induced apoptosis up to 37%. Combining these two drugs, the apoptosis rate raised to 68%, a significant approval of synergistic apoptosis induction of Met and Lira. 

***VEGF, angiogenin, PDGF, and MMP-9 proteins’ expression alteration by Lira and Met***

**Figure 8:**
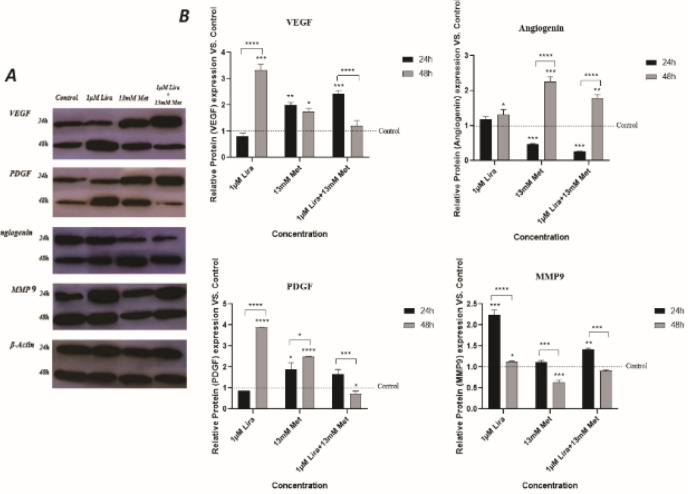
Effect of Metformin and Liraglutide alone and in combination on the expression level of angiogenic mediators in HUVECs after 24 hr and 48 hr time intervals. A) Representative immunoblot of VEGF, PDGF, Angiogenin, MMP-9, and β-actin. B) Relative protein expression of VEGF, PDGF, Angiogenin, and MMP-9. β-Actin protein was used as an internal control. Each band is representative of three independent experiments (*P<0.05, **P<0.01, ***P<0.001, and ****P<0.0001 were considered as the level of significance)

VEGF, angiogenin, PDGF, and MMP proteins as representatives of angiogenesis progress were analyzed through western blotting in the previously explained 4 groups (Figure 8). The combinational group indicated a significant increase in VEGF level at 24 hr, while its expression decreased after 48 hr compared with drugs alone. However, compared with the control, there were no significant changes in VEGF levels at 48 hr. The same procedure was also seen in PDGF. The following protein is angiogenin which is presented contradictorily. After 24 hr, its expression level decreased when Lira and Met combined, while it showed high expression when cells were exposed to drugs for 48 hr. In addition, we also examined MMP-9. As the results represent, although Lira raised the MMP-9 expression more than two times after 24 hr exposure, there were no significant changes at 48 hr. The same pattern was acquired for Met, in which the MMP-9’s expression decreased after 48 hr compared with 24 hr. Finally, the combination of Lira and Met increased protein expression at 24 hr, whereas no significant change was made after 48 hr compared with control. 

**Table 1:** CI scores for the combination of Metformin and Liraglutide at 24 hr drug exposure

*N*	*Metformin (mM)*	*Liraglutide (µM)*	*Effect*	*CI*
1	3.0	1.0	0.2765	0.32797 *
2	3.0	10.0	0.484	3.22394
3	3.0	20.0	0.2495	1.09666
4	3.0	40.0	0.188	1.27280
5	6.0	1.0	0.3463	0.45686 *
6	6.0	10.0	0.2197	1.10640
7	6.0	20.0	0.3482	2.35448
8	6.0	40.0	0.445	9.33764
9	13.0	1.0	0.4245	0.69184 *
10	13.0	10.0	0.1955	2.40267
11	13.0	20.0	0.4525	5.28482
12	13.0	40.0	0.5325	18.9217
13	20.0	1.0	0.2346	2.51540
14	20.0	10.0	0.2458	2.65372
15	20.0	20.0	0.2867	2.85014
16	20.0	40.0	0.1843	4.42784

**Table 2:** CI scores for the combination of Metformin and Liraglutide at 48 hr drug exposure

*N*	*Metformin (mM)*	*Liraglutide (µM)*	*Effect*	*CI*
1	3.0	1.0	0.5159	0.34240 *
2	3.0	10.0	0.4403	4.68004
3	3.0	20.0	0.4896	5.30593
4	3.0	40.0	0.2688	148.186
5	6.0	1.0	0.4542	0.80180 *
6	6.0	10.0	0.6642	0.47464 *
7	6.0	20.0	0.6912	0.59816 *
8	6.0	40.0	0.6862	1.15399
9	13.0	1.0	0.7652	0.15374 *
10	13.0	10.0	0.7033	0.43050 *
11	13.0	20.0	0.7356	0.44574 *
12	13.0	40.0	0.6844	1.31494
13	20.0	1.0	0.5184	1.17854
14	20.0	10.0	0.6889	0.62666 *
15	20.0	20.0	0.6539	1.24541
16	20.0	40.0	0.4946	10.8371

## Discussion

Angiogenesis is the formation of new capillary blood vessels from pre-existing vasculature, essential to embryonic growth and development. It is a normal physiological process throughout life for wound healing, bone repair and regeneration, post-ischemic tissue restoration, stress response, diabetic retinopathy, age-related macular degeneration, and thickening of the endometrium during the menstrual cycle. Angiogenesis is also involved in pathological processes and plays a crucial role in tumor growth, progression, and metastasis (21, 22).

Today, due to the crucial importance of the angiogenic switch during tumor growth, efforts to turn inhibition of tumor angiogenesis into an effective anti-cancer strategy have attracted much attention (22). Since angiogenesis primarily depends on the endothelial cells’ activation, migration, and proliferation, most angiogenic modulation approaches focus on the function of endothelial cells during vasculogenesis (23).

Accordingly, studies have been conducted on anti-diabetic drugs such as Met and Lira, which possess anti-inflammatory, cardiovascular-protective, and tumor-suppressive activities beyond their effect on glucose metabolism. These studies investigated the effects of these drugs on the risk of CVD, angiogenesis, vascular endothelial cell proliferation, and tumor extension.

For example, Goldberg (24) showed that treatment with Met in diabetes reduces CVD risk by lowering inflammation in these people. Krasner *et al*. (25) concluded from their study that Lira has anti-inflammatory effects on aortic endothelial progenitor cells due to increased intracellular calcium, leading to increased CaMKKB and AMPK activity. Other studies by Tomimoto *et al*. (26) and Kamarudin *et al*. (27) also showed that Met has anti-proliferative effects on vascular smooth muscle cells and inhibits the growth of tumors and intestinal polyps. In their study on the angiogenic properties of Lira as a GLP-1 analog and the mechanism involved, Langlois et al. (28) concluded that the beneficial effects of the drug on the islets of Langerhans appeared to be linked to its angiogenic properties. Similar results were obtained by researchers (5), who studied the promotion of the angiogenic ability of HUVECs by Lira. Arakawa *et al*. (29) also noted the anti-inflammatory effects of GLP-1 analogs in their study. In addition, after conducting their investigations, researchers (30) claimed that Lira improves the risk factors for CVD in diabetic patients, suggesting that this may be due to its effect on increasing endothelial cell proliferation. Ke *et al*. (31) decided to investigate the simultaneous effects of Met and Lira on vascular function in lipotoxicity-induced endothelial dysfunction. They have used Palmitic acid (PA) because it is usually found in the Western diet. PA increases ROS production, reduces NO level, and down-regulates p-eNOS protein expression. Their results indicated a much more significant impact on preventing endothelial cell dysfunction in the concurrent use of these two drugs compared with when they were used separately. Their results showed the protective effect of the two on blood vessels. 

Overall, many studies in recent years have confirmed the anti-inflammatory, tumor-suppression, and myocardial protection effects of these compounds.

Combining treatment with Met and Lira was more effective in preventing cell growth, proliferation, and migration than a single treatment. However, in the study of the effect of these two drugs on the expression of essential proteins in angiogenesis, the results did not meet expectations, and in general, no significant change was observed except for angiogenin.

Results showed that the synergistic inhibitory effect of two drugs on HUVEs cell proliferation was significant (75%) after 48 hr drug exposure. The anti-proliferative effect of Met has been shown in several tumor cell lines and endothelial cells due to cyclin D1 down-regulation (32-34).

 In addition, either Lira or Met alone had a marked tendency to inhibit the motility of endothelial cells as a hallmark of angiogenesis (42% and 39%), and almost a complete inhibition (97 %) was demonstrated in combinational use, after 48 hr treatment. While treatment with Met did not affect apoptosis contrary to the control group and Lira induced apoptosis up to 37%, after combining these two drugs, the apoptosis rate raised to 68%, which was a significant approval of synergistic apoptosis induction of Met and Liraa. 

The combinational group indicated a significant increase in the level of VEGF and PDGF at 24 hr, and compared with the control, there were no significant changes in VEGF and PDGF levels at 48 hr. VEGF and PDGF are pro-angiogenesis factors that exert biological effects by binding to their tyrosine kinase receptors expressed on vascular endothelial cells. Previously it was indicated that Lira did not increase VEGF protein expression in HUVECSs with 100 nmol/l of liraglutide for 24 hr (35). However, extending the treatment time to 48 hr significantly increased its expression. Di *et al*. showed that Lira stimulates the angiogenic potential of HUVECs by activating the JAK2/STAT3 pathway after 48 hr exposure (5). Our results are consistent with these. A paradoxical effect of Met on angiogenesis is indicated in various cellular contexts. Researchers found interesting results during evaluation of Met effects on HUVECSs cells. Their results demonstrate transient up-regulation of VEGF protein expression after 24 hr by Met (10 mM). But by extension of time, Met can inhibit capillary-like tubes formation by endothelial cells in an AMPK dependent manner. 

Migratory properties of endothelial cells are increased by VEGF-induced ERK activation (36). Met has been demonstrated to inhibit ERK pathway activation. Their results indicate that Met inhibits VEGF-induced ERK1/2 activation dependent on the AMPK. Our finding showed that after 48 hr, Met effects on VEGF decreased, and subsequently, VEGF-induced ERK activation was hampered, and migration significantly increased. However, Lira increased VEGF protein expression after 48 hr but is probably not sufficient to restore VEGF and PDGF protein expression in the presence of Metformin.

After 24 hr, angiogenin expression level decreased when Lira and Met combined, while it showed highly expressed when cells were exposed to drugs for 48 hr. The combination of Lira and Met increased MMP-9 expression at 24 hr, whereas no significant change was made after 48 hr compared with control. Angiogenin undergoes nuclear translocation in endothelial cells; this stage has a critical role in cell proliferation. It was previously demonstrated that angiogenin can stimulate ERK1/2 and phospholipase C pathways (37). Our results showed Met decreased angiogenin protein expression in HUVECs, after 24, but not after 48 hr exposure. A study on hepatocellular carcinoma indicated that Met decreased only angiogenin expression out of 20 angiogenesis-related proteins (38). As well as angiogenesis, angiogenin can promote cell growth (39).

Since either Met or Lira has vascular protective and anti-tumor effects, it could be reasonably hypothesized that a combination of both drugs might also have a synergistic effect on these processes. Similar results in confirming the anti-proliferative effects of Met on vascular smooth muscle cells have been found in other studies by Tomimoto *et al*. (26) and Kamarudin *et al*. (27). The results of this study were also consistent with the results of the study of Wang *et al*. (40). They studied the inhibitory effect of tumor angiogenesis using Met. This study showed that even in the presence of angiogenesis stimulants, Met inhibits angiogenesis. They said that Met also significantly reduces the amounts of HER2 and VEGF proteins and the density of tiny blood vessels. Besides, the results of a study conducted by Liu *et al*. (35) on the effect of Lira on angiogenesis in HUVECSs showed that treatment of cells with Lira increased the expression of VEGF and HIF in them. Another study (41) that examined the effect of GLP-1 analogs on growth inhibition of vascular smooth muscle cells showed that GLP-1 analogs inhibit PDGF expression that induces proliferation, differentiation, and migration of these cells.

On the contrary, Liu *et al*. (42), which studied the effects of Lira and Met alone or combined treatment on the cardiac function in type-2 diabetes patients, implied that the Lira mono-therapy showed more significant results than either Met alone or a combination of Lira and Met on cardiovascular function. In a very close study investigating the synergistic anti-tumor effect of these two drugs on pancreatic cancer cells, Lu *et al*. (18) suggested that Lira in combination with Met has a synergistic anti-tumor effect on the pancreatic cancer cells. Finally, based on the results of this study and evidence from other similar studies, it can be hypothesized that the synergistic anti-tumor effect of Lira and Met may be due to their interactive action on the GLP-1 receptor or activation of the AMPK signaling pathway in the studied cells.

## Conclusion

This study showed that combination therapy with Lira and Met could effectively reduce cell proliferation, induce cell apoptosis, and inhibit cell migration in the HUVECS cell line. This study provides evidence to support ​​using Met in combination with Lira as a treatment option for patients with type-2 diabetes and cancer. 

## Authors’ Contributions

HM, PT, AS, and SE Designed the experiments; SE, RS, NMD, and HJ Performed experiments and collected data; HF, HM, and AS Discussed the results and strategy; HM and PT Supervised, directed, and managed the study; AS, SE, NMD, RS, PT, HJ, HF, and HM Approved the final version to be published.

## Funding

This investigation was supported by Deputy of Research, Iran University of Medical Sciences, Tehran, Iran (grant no. 98-4-56-16746).

## Conflicts of Interest

The authors declare that there are no conflict of interests regarding this publication.
